# Sustainable carotenoid production using amylaceous agro-industrial byproducts: process efficiency and environmental assessment

**DOI:** 10.1186/s40643-026-01086-5

**Published:** 2026-06-13

**Authors:** Thércia Rocha Balbino, Salvador Sánchez-Muñoz, Stephanie Custódio Inácio, Gabriele Campelo Almeida, Ana Cláudia Dias, Júlio César Santos, Silvio Silvério da Silva, Jorge Fernando Brandão Pereira

**Affiliations:** 1 Laboratory of Bioprocesses and Sustainable Products, Department of Biotechnology, Engineering School of Lorena, University of São Paulo (EEL- USP), Estrada Municipal do Campinho, s/n - Pte. Nova, Lorena, SP 12602- 810 Brazil; 2 Centre for Environmental and Marine Studies (CESAM), Department of Environment and Planning, University of Aveiro, Campus Universitário de Santiago, 3810-193 Aveiro, Portugal; 3 Laboratory of Biopolymers, Bioreactors, and Process Simulation, Department of Biotechnology, Engineering School of Lorena, University of São Paulo (EEL-USP), Estrada Municipal do Campinho, s/n - Pte. Nova, Lorena, SP 12602-810 Brazil; 4 Department of Chemical Engineering, Faculty of Sciences and Technology, University of Coimbra, CERES, 3030-790 Coimbra, Portugal

**Keywords:** Bran, Hydrolysis, Pigments, Renewable feedstocks, Life cycle assessment

## Abstract

Agro‑industrial brans are abundant residues that can be valorized as low‑cost feedstocks for microbial bioprocesses within a circular‑economy framework. Here, we investigated the use of corn, soybean, rice and wheat bran hydrolysates as sole nutrient sources (no detoxification and no supplementation) for carotenoid production by the yeast *Rhodotorula mucilaginosa*. After standardizing total fermentable sugars to 10–12 g/L, the yeast successfully consumed mixed sugars and produced biomass and carotenoids in all hydrolysates. Rice bran hydrolysate led to the highest carotenoid titer (28.41 ± 0.23 mg/L) and the highest specific carotenoid content (1.11 ± 0.05 mg_carotenoids_/g_dry cells_), whereas soybean bran hydrolysate favored biomass formation (29.74 ± 0.18 g/L). UV–Vis/FTIR analyses of the pigment-rich extract showed spectral features consistent with carotenoids (absorption maxima at 480–490 nm and characteristic FTIR bands), supporting the qualitative identification of a carotenoid mixture. Finally, a cradle‑to‑gate life cycle assessment (LCA) showed that the RBH scenario presented the lowest impacts across the evaluated categories, with electricity demand during fermentation as the main hotspot. Overall, rice bran emerges as a promising residue for sustainable carotenoid bioproduction coupling process performance and environmental benefits. This study highlights the valorization of agro-industrial residues as an efficient strategy to reduce production costs and environmental impact, contributing to the development of more sustainable biotechnologies for carotenoid synthesis and beyond.

**Graphical abstract:**

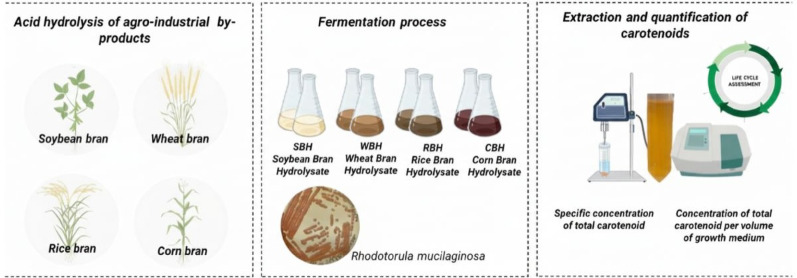

**Supplementary Information:**

The online version contains supplementary material available at 10.1186/s40643-026-01086-5.

## Introduction

The increasing environmental impacts associated with fossil fuels exploitation and the finite nature of petroleum-derived resources have intensified global interest in products obtained from alternative and renewable sources. (Ashokkumar et al. 2022; Antar et al. 2021). In this context, biorefineries have emerged as a cornerstone of the circular bioeconomy, as they enable the integrated and efficient conversion of renewable biomass into fuels, chemicals, and high value bioproducts while minimizing waste generation and resource losses (Mujtaba et al., 2023). A key challenge in biorefinery implementation lies in the cost, availability, and sustainability of feedstocks, which directly influence both economic and environmental performance.

The agro-industrial sector generates vast amounts of residual biomass, estimated at approximately 140 billion metric tons annually worldwide, most of which remains underutilized or is disposed of with limited value recovery. (UNEP, 2009; Koul et al. 2022). From a circular economy perspective, the valorization of these by-products as renewable feedstocks represents an effective strategy to close material loops, reduce waste, and replace fossil-based resources in industrial production chains. In particular, agro-industrial residues can serve as low-cost and nutrient-rich substrates for microbial bioprocesses, thereby enhancing both the economic viability and environmental sustainability of biorefineries.

Grain and cereal crops such as wheat, rice, corn, and sugarcane are among the main contributors to global agro-industrial biomass production (FAO STAT, 2022). During grain processing, large quantities of bran are generated as by-products. This biomass fraction is especially attractive for biotechnological applications, as it is rich in carbohydrates and starch (carbon sources), proteins (nitrogen sources), and essential minerals (micronutrients) (Anderson and Simsek 2019; Balbino et al. 2023). Owing to this balanced nutrient composition, bran represents an excellent substrate for the cultivation of microorganisms aimed at producing industrially relevant bioproducts, including pigments, proteins, and enzymes (Balbino et al. 2023; Spaggiari et al. 2021).

To efficiently exploit the nutritional potential of bran, appropriate pretreatment and conversion strategies are required to render sugars and nutrients accessible for microbial metabolism. Dilute-acid hydrolysis is a widely applied and industrially relevant approach that enables the release of fermentable sugars and soluble nutrients while maintaining operational simplicity. Importantly, this method allows the direct use of neutralized hydrolysates in fermentation, eliminating the need for acid recovery or extensive downstream processing steps that are typically associated with concentrated acid hydrolysis. By reducing process complexity, reagent consumption, and waste generation, dilute-acid hydrolysis contributes to the overall sustainability and economic feasibility of the bioprocess (Zhou et al. 2021).

Beyond feedstock availability, the success of a biorefinery depends on its capacity to generate a diverse portfolio of products, particularly high-value biomolecules that can improve process economics and reduce reliance on fossil-based alternatives (Yuan et al. 2021). While biofuels remain an important output, the growing environmental and market demands have stimulated interest in biotechnological products with enhanced functionality and lower environmental footprints. Among these, several high-value compounds derived from microbial processes have demonstrated superior performance in terms of efficiency and environmental positive impact (Medeiros et al. 2022).

Natural pigments are particularly promising targets for biorefinery-based production. In addition to their coloring properties, microbial biopigments exhibit a wide range of biological activities, including antioxidant, antimicrobial, cytotoxic, antimalarial, anticancer, and antitumor effects (Sharma et al. 2024). Carotenoids, a class of lipid-soluble pigments, are especially valued for their strong antioxidant capacity and are widely applied in the food, feed, cosmetic, and pharmaceutical industries (Foong et al. 2021; Díaz-Ruiz et al. 2023). The global carotenoids market is projected to reach USD 1.84 billion by 2024, with a compound annual growth rate of 4.64% between 2024 and 2029 (Mordor Intelligence Research & Advisory, 2023). Yeasts are particularly attractive producers of carotenoids due to their rapid growth, metabolic versatility, low production of toxic by-products, and ability to assimilate both C5 and C6 sugars, features that collectively reduce processing time and operational costs (Paul et al. 2023; Balbino et al. 2023).

In this study, we investigated the potential of corn, soybean, rice, and wheat bran hydrolysates (CBH, SBH, RBH, and WBH, respectively) as low-cost and renewable substrates for carotenoid production by *Rhodotorula mucilaginosa*. The proposed bioprocess was evaluated not only in terms of biopigment production but also through a life cycle assessment (LCA) approach, considering impacts related to global warming, ozone formation, terrestrial acidification, mineral resource scarcity, and fossil resource scarcity. By employing renewable feedstocks and avoiding intermediate processing steps, such as hydrolysate detoxification, nutrient supplementation, and acid recovery, this strategy aligns with the principles of green chemistry and the circular bioeconomy. As a result, it contributes to reducing resource consumption, waste generation, and environmental impacts, while enhancing the economic and environmental sustainability of pigment biorefineries and other bioprocesses based on grain and cereal by-products.

## Materials and methods

### Dilute-acid hydrolysis of grains by-products

Corn, soybean, rice and wheat brans were provided by local suppliers (Guaratinguetá, SP, Brazil). Prior to hydrolysis, the raw materials were classified using standard Tyler sieves (20/+28 mesh) to obtain a homogeneous particle‑size fraction, which was stored at 4 °C until use. The brans were then subjected to dilute-acid hydrolysis under the standard conditions established by Ayadi et al. (2019) and Martiniano et al. (2020). Dilute‑acid hydrolysis was carried out in an autoclave with 2% (v/v) sulfuric acid and 15% (w/v) total solids loading at 121 °C for 60 min. After hydrolysis, the liquid and solid fractions were separated via vacuum filtration. The liquid fractions (corn bran hydrolysate, CBH; soybean bran hydrolysate, SBH; rice bran hydrolysate, RBH; and wheat bran hydrolysate, WBH) were recovered, and their pH was adjusted when required prior to fermentation. Hydrolysates were characterized for sugars (glucose, xylose and arabinose) and potential inhibitors (furfural and 5‑HMF) by high-performance liquid chromatography (HPLC), and for total soluble proteins by spectrophotometry, as described below. Hydrolysates were stored at − 4 °C until use.

### Yeast-derived carotenoid production

#### Microorganism and inoculum preparation

Yeast *Rhodotorula mucilaginosa* was used to produce carotenoids. To activate the yeast, cells from the stock culture were transferred to Erlenmeyer^®^ flasks (125 mL) containing 30 mL of medium with commercial glucose (YM) (composition (g/L): glucose (30), peptone (5), yeast extract (3), and malt extract (3)). The flasks were incubated in an orbital shaker at 30 °C, 300 rpm for 18 h. The cells were recovered by centrifugation at 3000 xg for 15 min, washed, and resuspended in 0.1% (w/v) peptone aqueous solution. The cells were utilized as inoculum for the subsequent fermentation processes.

#### Hydrolysates as a growth medium for carotenoid production

Considering the different concentrations of sugar in the four hydrolysates, the concentration of sugars (sum of glucose, xylose, and arabinose) in CBH, RBH, and WBH was standardized to approximately 10–12 g/L by simply adding the right amount of water, guaranteeing the minor concentration obtained after soybean bran hydrolysis. This “sugar standardization” aimed to obtain comparable results between hydrolysates. The initial pH of each hydrolysate was adjusted to 5.5 by adding sodium hydroxide micro-pearls. Erlenmeyer^®^ flasks (250 mL) containing 60 mL of CBH, SBH, RBH, or WBH were sterilized at 121 °C for 15 min in an autoclave. After sterilization, the flasks were inoculated with activated yeast cells and incubated at 30 °C, 300 rpm for 72 h. Samples were collected every 2 h up to 10 h and after 24, 48, and 72 h to determine the cellular biomass concentration and sugar consumption.

### Cell disruption and carotenoid extraction

Because carotenoids are produced intracellularly, a proper cell disruption process and biopigment extraction were applied to the samples. The harvested pigmented cell pellets were washed three times, resuspended in 2 mL distilled water, and dried at 60 °C for 24 h. 0.1 g of dry cells were resuspended in 10 mL of 2 mol/L NaOH aqueous solution and incubated in a water bath at 65 °C for 10 min. The cells were centrifuged (3000 x*g* for 15 min) and frozen overnight. The pellets were resuspended in 10 mL methanol: acetone solution (7:3, v/v) and homogenized by ultrasonication with cycles of 20 s ON/5 s OFF for 10 min to achieve complete cell disruption and the release of intracellular carotenoids. The cell debris was separated by centrifugation (3000 x*g* for 15 min) and the colored supernatants (carotenoid-rich extracts) were collected.

### Life cycle assessment (LCA)

The life cycle analysis (LCA) was conducted to compare the potential environmental impacts associated with yeast-derived carotenoid production using hydrolysates from corn, soybean, rice, and wheat bran, to identify the scenarios with the lowest impacts.

The environmental profile of the procedure proposed in this study was evaluated using LCA methodology according to the ISO 14,040 and ISO 14,044 standards (ISO 2006a, b). Data on the impacts of producing the materials and electricity consumed were mostly sourced from the Ecoinvent database version 3.9.1. (Wernet et al. 2016). However, as data were not available for corn bran and soybean bran production, they were obtained from the World Food LCA database version 3.5 and Agribalyse version 3.1, respectively (Nemecek et al. 2019; Asselin-Balençon et al. 2022). The SimaPro software version 9.5.0.2 was used to model the process (Table S1) and to calculate the environmental impacts. The system boundary adopted was cradle-to-gate, from bran hydrolysate preparation to fermentation. The impacts were estimated by applying the ReCiPe 2016 method from the hierarchist perspective (Huijbregts et al. 2017). Based on data availability, the following impact categories were selected: global warming (equivalent to the carbon footprint), ozone formation - human health, terrestrial acidification, mineral resource scarcity, and fossil resource scarcity.

### Analytical methods

#### Cell biomass concentration

Yeast cell concentrations were determined using a spectrophotometer. Absorbances were converted to concentration (g/L) using a previously established standard curve. The maximum specific growth rate (µmax, h^− 1^) was determined by linear regression between the optical density (600 nm) obtained from the exponential growth phase and time (h).

#### Determination of sugar, furan derivate, and phenolic compounds

Glucose, xylose, and arabinose were quantified using an HPLC Agilent 1200 series (Agilent Technologies Inc., USA) equipped with a refractive index detector (RID-6 A) and HPX-87 H (300 × 7.8 mm) column (Bio-Rad, USA). The analysis was performed under the following conditions: 45 °C column temperature, 0.01 N H_2_SO_4_ as the mobile phase, 0.6 mL/min flow rate, and 20 µL injection volume.

Furan derivatives (furfural and 5-hydroxymethylfurfural (5-HMF) and phenolic compounds (4-hydroxybenzoic acid, ferulic acid, gallic acid, p-coumaric acid, vanillic acid, pyrocatechol, syringaldehyde, and vanillin) were analyzed according to Skendi et al. (2017) using a Zorbax C18 column (Agilent, Santa Clara, CA, USA) at 30 °C with a gradient of 1% (v/v) acetic acid in water (A), acetonitrile (B), and methanol (C) at a flow rate of 1.3 mL/min. The respective analytes were detected using a UV detector at wavelengths of 260, 280, and 320 nm.

#### Determination of the total soluble protein concentration

The concentration of total soluble proteins was determined according to the method described by Lowry et al. (1951). Approximately 5 ml of reagent A (48 ml of 2% sodium carbonate in 0.1 n sodium hydroxide, 1 ml of 0.5% copper sulfate, and 1 ml of 1% sodium potassium tartrate) was added to the sample and kept aside for 15 min. 0.5 ml of freshly prepared reagent B (Folin-Ciocalteu solution: water, 1:1) was added and mixed. The test tubes were then incubated for 30 min in the dark. Measurements were taken at 660 nm, according to the absorption spectrum of the colored reaction product, using an Eppendorf Biospectrometer^®^ fluorescence. A standard reference curve was prepared using bovine serum albumin (Sigma-Aldrich) at 40 to 400 µg/mL concentrations.

#### Carotenoids analysis and quantification

Total carotenoids were quantified spectrophotometrically as β‑carotene equivalents following a calibration approach commonly used for yeast carotenoid extracts (Manimala and Murugesan 2017). Pigments extracted from cell pellets using methanol: acetone (7:3, v/v) and the clarified extracts were scanned from 350 to 700 nm to identify the maximum absorption. Quantification was performed at 483 nm (within the 480–490 nm region characteristic of *Rhodotorula* carotenoids), using a β‑carotene standard curve (Sigma‑Aldrich) prepared in the same solvent mixture at 1–60 µg/mL to minimize standard consumption. Spectrophotometric analyses were performed on a Thermo Scientific^®^ UV-Vis spectrophotometer (model Genesis 10 S, China).

Carotenoid concentration in the extract was first obtained as C (mg_carotenoids_/L_solvent_) from the calibration curve and then used to calculate (i) the specific carotenoid content (SC, mg_carotenoids_/g_dry cells_) and (ii) the carotenoid titer per culture volume (TC, mg/L) according to Eqs. (1)–(2).

1 $$\:\mathrm{S}\mathrm{C}\:=\:\frac{(C\:\times\:\:Vs)}{(DW\:\times\:\:Vc)}$$

2 $$\:\mathrm{T}\mathrm{C}\:=\:\frac{C\:\times\:\:Vs}{Vc}$$

where C is total carotenoids in the extract (mg/L solvent); Vs is the solvent volume used for extraction (L); DW is the dry cell mass used for extraction (g); and Vc is the culture volume (L).

### Statistical analysis

Data were analyzed using STATISTICA (StaSoft, Inc., Oklahoma, USA) and are presented as mean ± standard deviation (SD). Means were tested for significant differences with 95% confidence through a one-way analysis of variance (ANOVA) followed by Tukey’s post hoc test. The level of significance was set at *p* < 0.05.

## Results and discussion

### Bran hydrolysates characterization

Cereal and grain cultivation, including corn, soybean, rice, and wheat, are globally widespread agricultural practices that generate substantial amounts of by-products when processed at an industrial scale (Kaur et al. 2023). Consequently, evaluating the potential of agro-industrial by-products as low-cost sources of carbon and nutrients for the production of high-value-added compounds is of significant interest. However, despite their nutritional potential, the effective utilization of these biomasses requires the release of fermentable sugars and nutrients from the complex plant matrix.

To achieve this, dilute-acid hydrolysis was applied to corn, soybean, rice, and wheat bran, yielding corn bran hydrolysate (CBH), soybean bran hydrolysate (SBH), rice bran hydrolysate (RBH), and wheat bran hydrolysate (WBH), respectively. Dilute-acid hydrolysis is a well-established and efficient method for the depolymerization of lignocellulosic and amylaceous materials, as it promotes acid penetration into plant cell walls and weakens the intermolecular interactions between cellulose, hemicellulose, and lignin. This process enhances the availability of sugars and nutrients for microbial assimilation (Lv et al. 2024). The resulting hydrolysates contained varying concentrations of pentoses, hexoses, proteins, as well as compounds that may inhibit microbial growth. Differences in sugar release among the hydrolysates are likely associated with variations in the proportions of starch, cellulose, and hemicellulose, as well as structural differences inherent to each type of bran.

The total sugar concentration (sum of glucose, xylose, and arabinose) varied markedly among the hydrolysates, ranging from 14 to 105 g/L. Specifically, CBH, SBH, RBH, and WBH contained 104.33 ± 0.36 g/L, 11.04 ± 0.34 g/L, 23.85 ± 0.45 g/L, and 43.37 ± 0.66 g/L, respectively. According to previous studies, corn bran typically contains approximately 12–77% carbohydrates, 11–32% starch, 10–28% cellulose, and 1–9% lignin (Philippini et al. 2021; Probst and Vadlani 2015; Rose et al. 2010). The relatively lower lignin content combined with higher carbohydrate, starch, and cellulose fractions in corn bran likely facilitated more extensive glucose release during acid hydrolysis, resulting in a hexose-rich hydrolysate (Zhou et al. 2021).

**Fig. 1:**
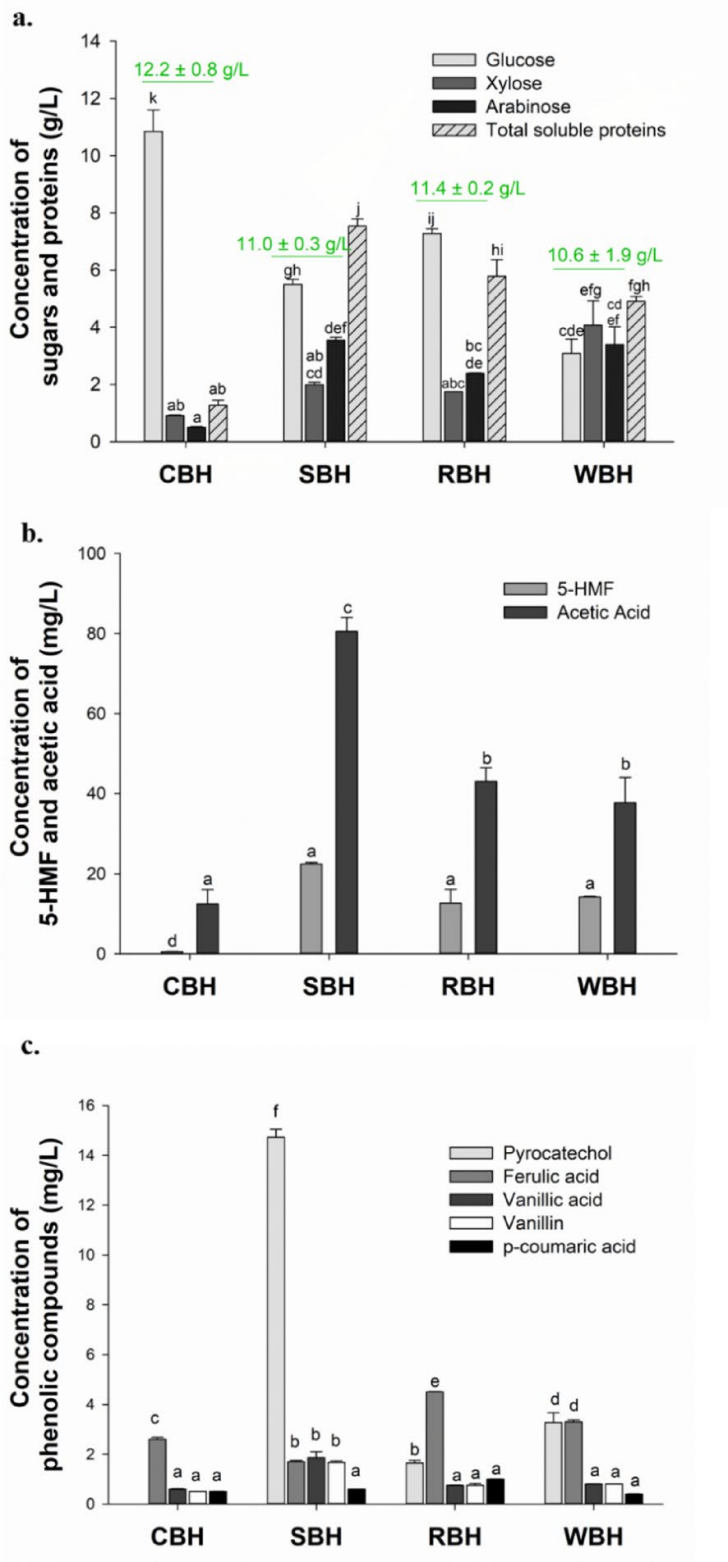
Composition of standardized corn bran hydrolysate (CBH), soybean bran hydrolysate (SBH), rice bran hydrolysate (RBH), and wheat bran hydrolysate (WBH). **(a**) Concentrations of sugars and total soluble proteins. Sugar concentration, expressed as the sum of glucose, xylose, and arabinose, was adjusted to 10–12 g/L. Values above the bars (highlighted in green) represent the mean ± standard deviation of the final sugar concentration after hydrolysate standardization. (**b**) Concentrations of acetic acid and 5-hydroxymethylfurfural (5-HMF). (**c**) Concentrations of phenolic compounds. Bars sharing the same letter within the same group are not significantly different according to Tukey’s test (*p* ≤ 0.05)

To enable a meaningful comparison of cell growth, sugar consumption, and carotenoid production across the different substrates, the total sugar concentration (sum of glucose, xylose, and arabinose) in all hydrolysates was adjusted to 10–12 g/L by dilution with water. This concentration corresponds to the lowest sugar content observed in SBH. Following standardization, the bran hydrolysates were characterized in terms of glucose, xylose, and arabinose concentrations, total soluble protein content, and the presence of microbial growth-inhibiting compounds, including organic acids, furan derivatives, and phenolic compounds. The corresponding results are presented in Fig. 1.

The standardized bran hydrolysates exhibited clear differences in glucose concentration, following the order CBH > RBH > SBH > WBH (Fig. 1a). In CBH, glucose reached 10.85 ± 0.75 g/L, accounting for approximately 88% of the total sugar content (sum of glucose, xylose, and arabinose), with only trace amounts of xylose and arabinose detected. Similarly, glucose was the predominant sugar in RBH and SBH, representing 63.7% and 49.7% of the total sugar content, respectively, although the relative differences between glucose/xylose and glucose/arabinose ratios were less pronounced than in CBH. In both RBH and SBH, arabinose concentrations exceeded those of xylose, with a more marked difference observed in SBH (32.1% arabinose and 18.0% xylose) compared to RBH (20.9% arabinose and 15.3% xylose). In contrast, WBH was characterized by xylose as the dominant sugar (4.07 ± 0.40 g/L), with comparable concentrations of glucose, xylose, and arabinose and a higher xylose-to-glucose ratio than the other hydrolysates. This profile is likely related to the higher hemicellulose content of wheat bran biomass (Ayadi et al. 2019).

Consistent with the sugar profiles, the total soluble protein content varied among the hydrolysates in the order SBH > RBH > WBH > CBH (Fig. 1a). However, the ratio between total sugars (carbon sources) and soluble proteins (nitrogen sources) showed relatively limited variation among SBH (1.46 ± 0.50), WBH (2.16 ± 0.54), and RBH (1.99 ± 0.18), whereas CBH exhibited a substantially higher carbohydrate-to-protein ratio (8.83 ± 0.89).

In addition to fermentable sugars, compounds capable of inhibiting microbial growth and metabolism may also be generated during the hydrolysis of lignocellulosic and amylaceous biomass. Acetic acid, for example, is formed through the deacetylation of acetylated pentosans and the hydrolysis of acetyl groups in hemicellulose (Świątek et al. 2020). Similarly, 5-hydroxymethylfurfural (5-HMF) can be produced through a sequence of reactions involving glucan hydrolysis, glucose isomerization to fructose, and subsequent fructose dehydration (Iris and Tsang 2017; Zhao et al. 2019). Among the standardized bran hydrolysates, SBH exhibited the highest acetic acid concentration (80.50 ± 3.53 mg/L), which was approximately twice that observed in RBH and WBH (43.00 ± 3.46 and 37.66 ± 3.35 mg/L, respectively) and about six times higher than that in CBH (12.50 ± 3.50 mg/L). In contrast, 5-HMF concentrations ranged between 12 and 20 mg/L in SBH, RBH, and WBH, while only trace amounts were detected in CBH (0.57 ± 0.06 mg/L) (Fig. 1b).

Phenolic compounds, which originate mainly from lignin degradation, may also be formed during acid hydrolysis of lignocellulosic and amylaceous biomass (Luo et al. 2021). Among the quantified phenolics, pyrocatechol was detected at higher levels in SBH (14.73 ± 0.31 mg/L) but was not detected in CBH (Fig. 1c). Ferulic acid was present in all hydrolysates, with concentrations ranging from 1.7 to 4.5 mg/L, and RBH exhibiting the highest content. Vanillic acid, vanillin, and p-coumaric acid were detected at lower concentrations across all hydrolysates (0.4–1.8 mg/L), with SBH showing the highest overall levels. It should be noted that the concentrations of inhibitory compounds in the standardized hydrolysates (Fig. 1b and c) were influenced by the dilution required to adjust sugar concentrations to 10–12 g/L. Due to its initially higher sugar content, CBH required greater dilution, resulting in lower concentrations of inhibitory compounds after standardization.

Given that growth medium composition strongly influences microbial growth kinetics, substrate utilization, and secondary metabolite production, the four standardized hydrolysates were subsequently evaluated as growth media for *Rhodotorula mucilaginosa* cultivation and carotenoid production.

### *Rhodotorula mucilaginosa* cultivation and carotenoid production using standardized bran hydrolysates

#### Sugar consumption

**Fig. 2:**
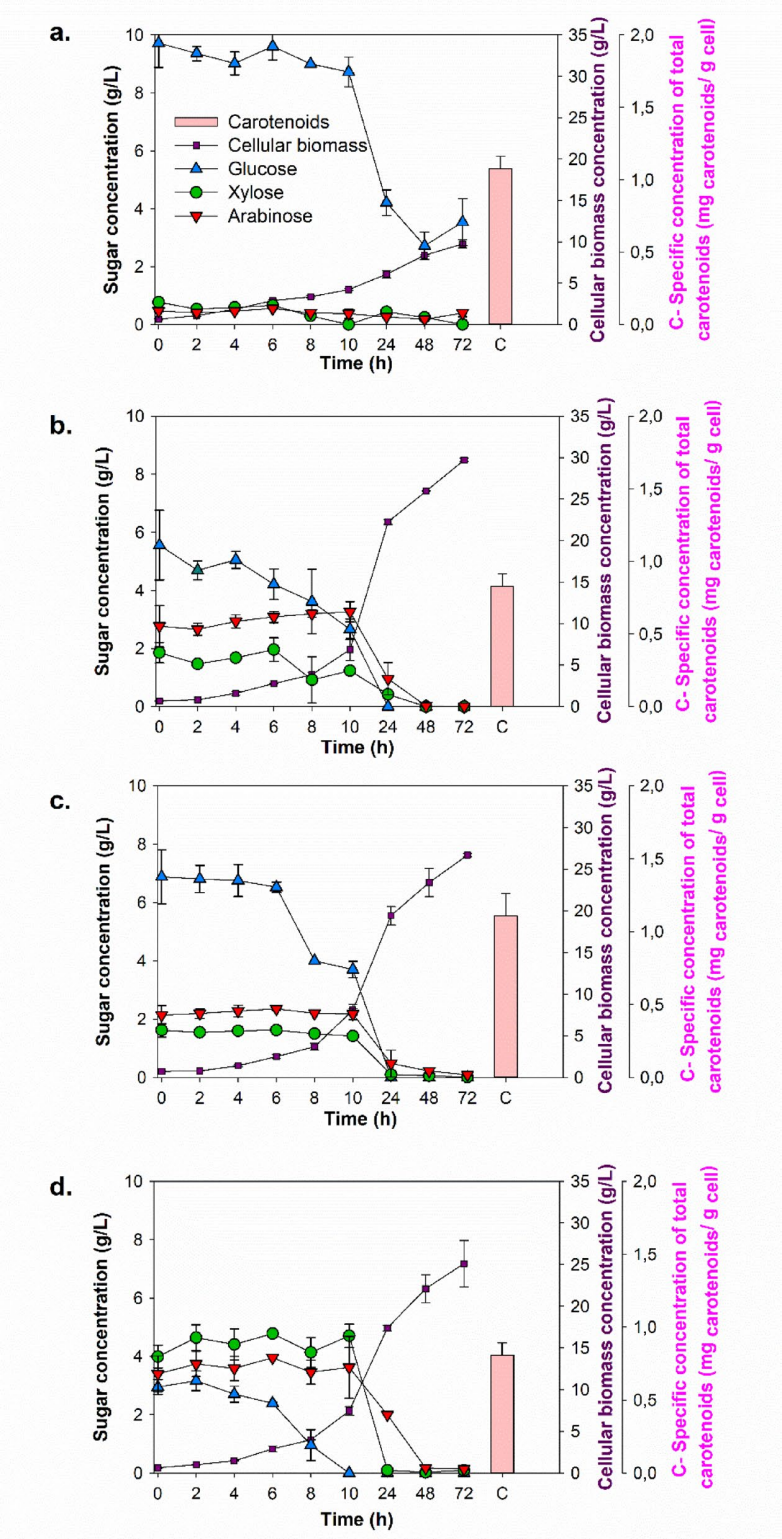
Sugar consumption, cell production, specific carotenoid content (mg_carotenoids_/g _dry cells_), and carotenoid concentration per culture volume (mg_carotenoids_/L_culture_) by *R. mucilaginosa* cultivated in (**a**) corn bran hydrolysate (CBH), (**b**) soybean bran hydrolysate (SBH), (**c**) rice bran hydrolysate (RBH), and (**d**) wheat bran hydrolysate (WBH)

*R. mucilaginosa* is known for its ability to assimilate a wide range of carbon sources, including both hexoses and pentoses (Hamidi et al. 2020). In this study, the consumption of glucose, xylose, and arabinose by *R. mucilaginosa* cultivated in standardized bran hydrolysates was evaluated (Fig. 2). Although sugar consumption patterns varied depending on hydrolysate composition, glucose was consistently the first sugar to be consumed under all cultivation conditions.

A distinct behavior was observed for cultures grown in CBH (Fig. 2a). In this case, glucose was not completely depleted after 72 h of cultivation, in contrast to the results obtained with the other hydrolysates. Approximately 3.0 g/L of residual glucose remained at the end of the fermentation. In addition, arabinose was not consumed, whereas xylose consumption occurred gradually between 10 and 72 h (Fig. 2a).

In contrast, SBH, WBH, and RBH supported more efficient sugar utilization by the yeast. Under these conditions, *R. mucilaginosa* was able to completely consume glucose, xylose, and arabinose within 72 h of cultivation. These results indicate that, despite standardized sugar concentrations, differences in hydrolysate composition significantly influenced sugar uptake kinetics and overall carbon utilization by *R. mucilaginosa.*

When WBH was used as the growth medium (Fig. 2d), glucose remained the preferred carbon source, whereas xylose and arabinose assimilation began only after glucose depletion, which occurred at approximately 10 h of cultivation. Under these conditions, xylose was consumed at a faster rate than arabinose. These differences in sugar utilization patterns can be attributed to variations in sugar composition, total soluble protein content, and the presence of microbial growth-inhibitory compounds in the hydrolysates, all of which may influence yeast metabolic activity.

Overall, the results demonstrate the ability of *R. mucilaginosa* to metabolize multiple sugars present in amylaceous hydrolysates. The observed differences in sugar consumption profiles underscore the importance of selecting appropriate by-product-based growth media to optimize yeast cultivation and carotenoid production, thereby improving bioprocess efficiency and overall yield.

#### Cell biomass and carotenoid production

Similar to the variations observed in sugar consumption, differences in cell biomass formation and carotenoid production were strongly influenced by the composition of the bran hydrolysates, which directly affect microbial growth and metabolite biosynthesis. Regarding biomass production by *R. mucilaginosa*, comparable growth patterns were observed when the yeast was cultivated in SBH, RBH, and WBH (Figs. 2b–d). Under these conditions, final cell biomass concentrations ranged from 25 to 30 g/L, and the maximum specific growth rate (µ_max_) varied between 0.25 and 0.27 (h^− 1^) (Table 1).

In contrast, cultivation in CBH resulted in significantly lower biomass production (9.75 g/L) and a reduced µ_max_ (0.16 h^− 1^). Moreover, yeast growth in CBH slowed markedly after approximately 10 h of cultivation, compared with the other standardized hydrolysates (Figure S1). These results indicate that, despite comparable initial sugar concentrations, differences in nutrient availability and hydrolysate composition had a pronounced impact on yeast growth performance.

Clear differences in biopigment accumulation were also observed among the hydrolysates, following the trend RBH ≈ CBH > SBH ≈ WBH (Table 1). RBH led to the highest specific carotenoid content (1.11 ± 0.05 mg_carotenoids_/g_dry cells_) and the highest carotenoid titer per culture volume (28.41 ± 0.23 mg/L). This was followed by SBH (24.55 ± 0.25 mg/L) and WBH (20.28 ± 0.51 mg/L), whereas CBH resulted in the lowest carotenoid concentration per culture volume (10.50 ± 0.31 mg/L). These variations are consistent with differences in the nutrient matrices of the brans, as higher soluble protein levels and more balanced carbon-to-nitrogen (C/N) ratios favor both yeast growth and carotenoid biosynthesis. Conversely, limited nutrient availability can restrict biomass formation and carotenogenesis. Therefore, substrate selection plays a critical role in determining carotenoid productivity, even when fermentable sugar concentrations are standardized.

**Table 1:** Cell biomass and carotenoid concentrations produced by yeast *R. mucilaginosa* cultivated in corn bran hydrolysate (CBH), soybean bran hydrolysate (SBH), rice bran hydrolysate (RBH), and wheat bran hydrolysate (WBH)

	CBH	SBH	RBH	WBH
Cell biomass concentration (g/L)	9.75 ± 0.52	29.74 ± 0.18	25.69 ± 0.16	25.11 ± 0.78
µ_max_ (h^− 1^)	0.16 ± 0.01	0.26 ± 0.02	0.27 ± 0.02	0.25 ± 0.04
Specific carotenoid content, SC (mg_carotenoids_/g_dry cells_)	1.08 ± 0.08	0.83 ± 0.09	1.11 ± 0.05	0.81 ± 0.08
Total carotenoid concentration, TC (mg/L)	10.50 ± 0.31	24.55 ± 0.25	28.41 ± 0.23	20.28 ± 0.51

Although CBH enabled the accumulation of relatively high specific carotenoid content (1.08 mg_carotenoids_/g_dry cells_), comparable to that obtained with RBH, its overall carotenoid titer was considerably lower due to reduced biomass formation. Thus, despite enhanced intracellular carotenoid accumulation, the use of CBH may result in lower process productivity, which is a critical limitation for industrial-scale implementation.

The most promising results were achieved using RBH, which supported both robust cell growth (25.69 g/L) and high specific carotenoid accumulation (1.11 mg_carotenoids_/g_dry cells_), indicating a balanced metabolic allocation between growth and secondary metabolite production. Consequently, RBH yielded the highest carotenoid concentration per unit volume (28.41 mg/L), highlighting the strong potential of rice bran as a feedstock for sustainable biopigment production.

The feasibility of using agro-industrial by-products as substrates for carotenoid production by *R. mucilaginosa* has been previously reported. For example, Manimala and Murugesan (2017) evaluated several alternative biomass sources, including rice bran, wheat bran, cassava bagasse, and various oil cakes. In that study, cassava bagasse resulted in the highest carotenoid production, while rice bran showed limited performance when used *in natura* and combined with synthetic media. In contrast, the higher productivity observed in the present study using RBH suggests that dilute-acid pretreatment plays a crucial role in enhancing sugar release and nutrient availability, thereby improving microbial metabolism and bioprocess performance. Moreover, dilute-acid hydrolysis allows the direct use of neutralized hydrolysates in fermentation without additional acid separation or recovery steps (Zhou et al. 2021). This strategy also eliminates the need for hydrolysate detoxification or nutrient supplementation, reducing reagent consumption and preventing the generation of undesirable waste streams, which collectively improves both the economic and environmental sustainability of the process.

**Fig. 3:**
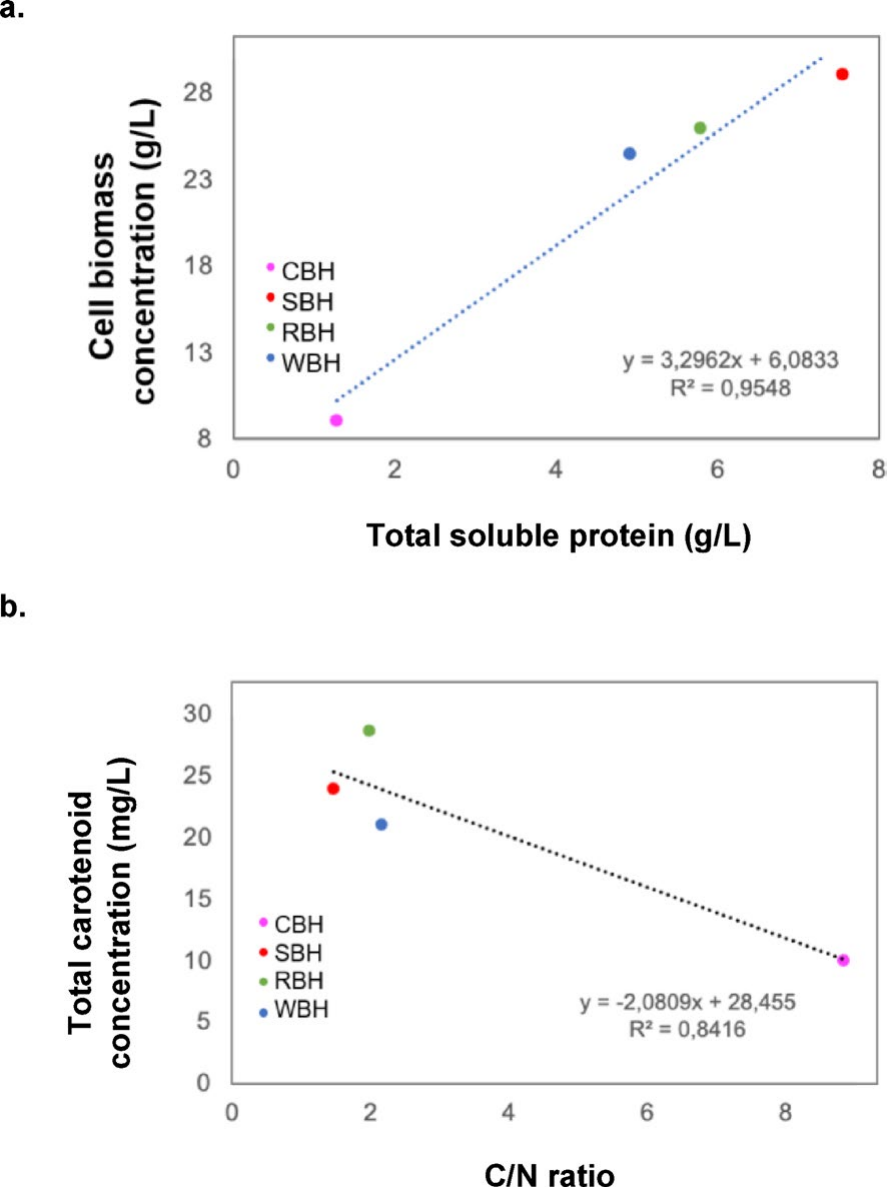
Correlation between (**a**) cell biomass concentration (g/L) and the total soluble protein content of the hydrolysates (g/L), and (**b**) total carotenoid concentration per culture volume (mg/L) and C_carbohydrate_/N_proteins_ ratio of the hydrolysates. Carotenoids were produced by *R. mucilaginosa* cultivated in corn bran hydrolysate (CBH), soybean bran hydrolysate (SBH), rice bran hydrolysate (RBH), and wheat bran hydrolysate (WBH)

To further elucidate the influence of hydrolysate composition on yeast performance, cell biomass production and carotenoid titers were correlated with total soluble protein content and C_carbohydrate_/N_protein_ ratios, respectively. As shown in Fig. 3a, cell biomass concentration was positively correlated with the soluble protein content of the hydrolysates. Given that total sugar concentrations were standardized to 10–12 g/L, the lower biomass production observed with CBH can be attributed to its reduced nitrogen availability and consequently higher C/N ratio.

Variations in carbon and nitrogen availability are known to influence metabolic flux distribution, potentially diverting carbon away from biomass formation or carotenoid synthesis (Gedela et al., 2022; Elfeky et al. 2019). Hydrolysates with C_carbohydrate_/N_protein_ rates close to 2 (SBH, WBH, and RBH) supported higher carotenoid production than CBH, which exhibited a ratio close to 8 (Fig. 3b). Achieving an optimal balance between microbial growth and carotenoid accumulation remains a key challenge in yeast-based biopigment production (Li et al. 2022). Under the evaluated conditions, RBH and SBH provided the most favorable balance; however, RBH stood out by enabling higher specific carotenoid accumulation.

In summary, these results demonstrate that *R. mucilaginosa* can effectively grow and produce carotenoids using CBH, SBH, RBH, and WBH as sole nutrient sources, without requiring hydrolysate detoxification or medium supplementation. By reducing processing steps, reagent consumption, and waste generation, this approach enhances the economic and environmental sustainability of the bioprocess, reinforcing the potential of agro-industrial by-products as feedstocks for next-generation pigment biorefineries.

#### Spectrophotometric and FTIR characterization of the carotenoid-rich extract

**Fig. 4:**
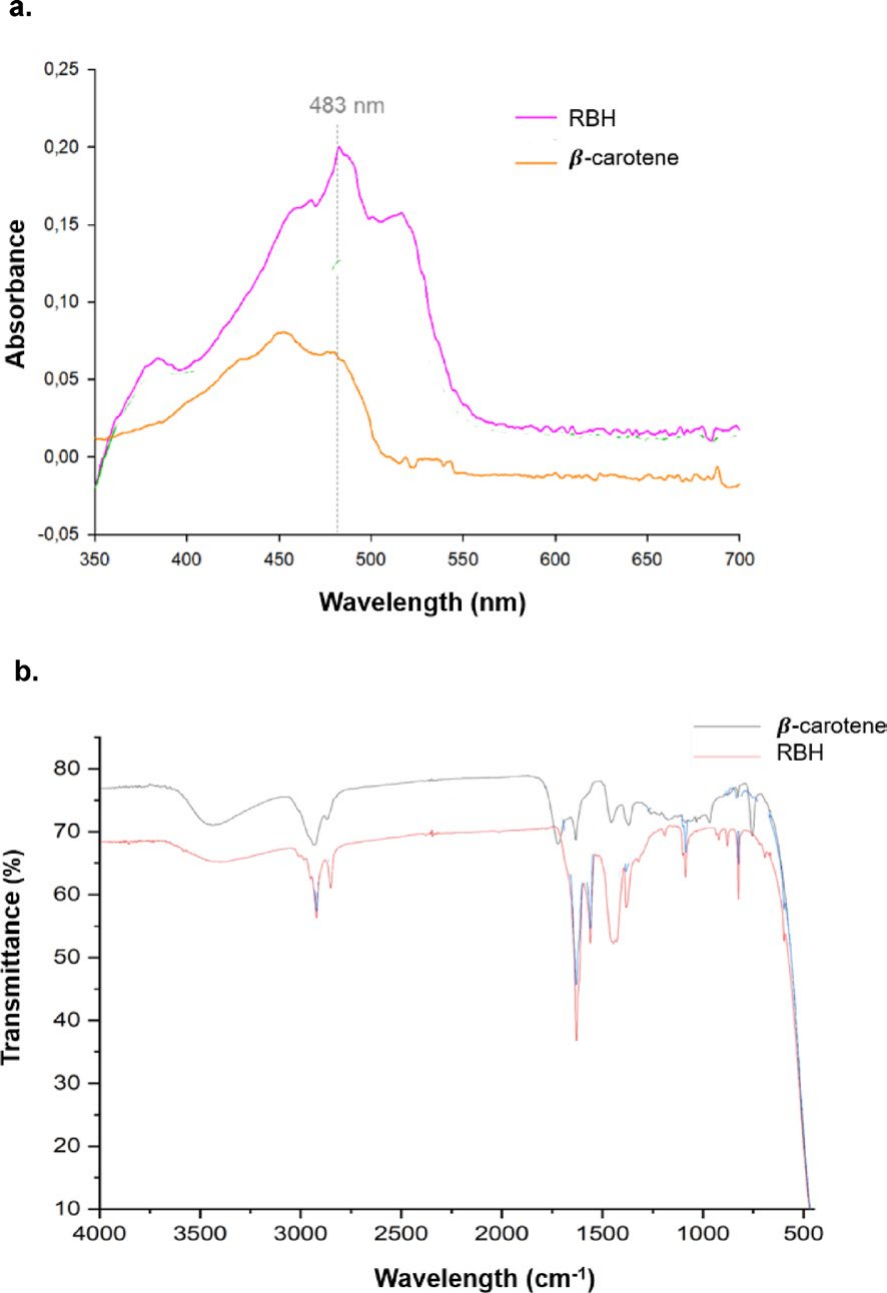
UV–Vis (**A**) and FTIR (**B**) spectra of the pigment-rich extract obtained from R. mucilaginosa cultivated in RBH and of a β-carotene standard. Samples were solubilized in methanol:acetone (7:3, v/v)

To qualitatively assess the carotenoid composition of the colored extract, a representative pigment-rich extract obtained from *R. mucilaginosa* cultivated in RBH was analyzed by UV–Vis spectrophotometry and FTIR spectroscopy (Fig. 4).

The UV-Vis spectrum exhibited absorption maxima between 480 and 490 nm (Fig. 4A), which are characteristic of microbial carotenoids. According to the literature (Mussagy et al. 2021), absorption peaks around 450 nm are associated with β-carotene, while peaks at approximately 484 nm and 490 nm correspond to torulene and torularhodin. Therefore, the observed profile supports the presence of a mixture of carotenoids whose relative proportions may vary with the microorganism and cultivation conditions.

FTIR spectra were recorded in the range of 4000–500 cm⁻¹. The β-carotene standard exhibited characteristic absorption bands at 2922 and 2848 cm⁻¹, attributed to C–H stretching of methyl and methylene functional groups, and a prominent band at 1630 cm⁻¹, associated with C = C stretching typical of conjugated double bonds in carotenoids. Additional peaks at 1441 and 1374 cm⁻¹ were assigned to C–H bending vibrations.

The carotenoid-rich extract showed similar bands at 2924 and 2849 cm⁻¹, consistent with the presence of carotenoid-like structures. However, additional bands not observed in the β-carotene standard were detected, such as a peak at 1558 cm⁻¹, likely associated with amide II vibrations (N–H bending and C–N stretching), indicating the presence of proteins. The band at 1085 cm⁻¹ may be attributed to C–O–C stretching in esters or C–C stretching in lipid and carotenoid chains. A band at 825 cm⁻¹¹, observed in both samples, corresponds to –CH₂ rocking vibrations typical of hydrocarbon chains.

Overall, FTIR analysis supports that the colored extract consists of a complex mixture of carotenoids, lipids, and proteins, reflecting the intracellular nature of pigment accumulation in yeast cells.

### Life cycle assessment

Life cycle assessment (LCA) has been widely applied to evaluate the environmental impacts of carotenoid production, particularly for microalgal systems producing high-value compounds such as astaxanthin and β-carotene. In contrast, studies addressing the environmental performance of yeast-based carotenoid production using renewable agro-industrial feedstocks remain scarce (de Oliveira et al. 2024). To address this gap, a comprehensive LCA was performed considering five midpoint impact categories: global warming, ozone formation (human health), terrestrial acidification, mineral resource scarcity, fossil resource scarcity. The objective was to compare four bioprocess scenarios and assess the sustainability of converting agro-industrial by-products into hydrolysates for microbial biopigment production within a biorefinery framework.

**Fig. 5:**
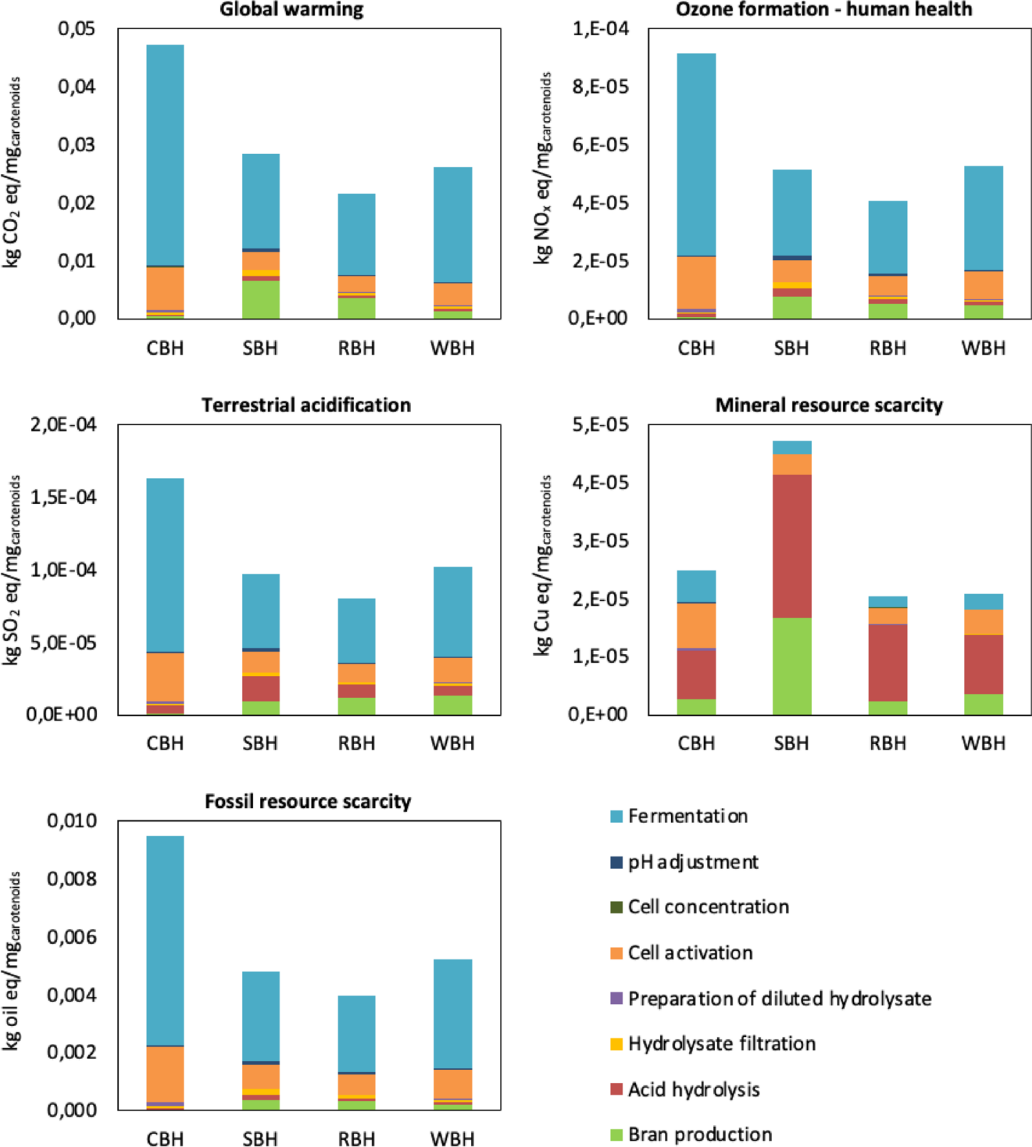
LCA results for corn bran hydrolysate (CBH), soybean bran hydrolysate (SBH), rice bran hydrolysate (RBH), and wheat bran hydrolysate (WBH)

Four scenarios were evaluated based on the use of different bran types as feedstocks: corn, soybean, rice, and wheat bran. In each case, approximately 58 g of bran were subjected to dilute-acid hydrolysis, producing 300 mL of concentrated hydrolysate with variable total sugar content (sum of glucose, xylose, and arabinose). After hydrolysis, the hydrolysates were diluted to standardize sugar concentrations and subsequently used growth media for fermentation. Prior to fermentation, *Rhodotorula mucilaginosa* cells were activated in YM medium and concentrated by centrifugation. The standardized hydrolysates were then inoculated, and fermentation was carried out for 72 h. Environmental impacts were calculated and expressed per mg of carotenoids, allowing a direct comparison among corn bran hydrolysate (CBH), soybean bran hydrolysate (SBH), rice bran hydrolysate (RBH), and wheat bran hydrolysate (WBH) (Fig. 5).

Across all evaluated scenarios and categories, the fermentation step was the dominant contributor to environmental impacts, mainly due to electricity consumption associated with agitation and temperature control. Similar environmental hotspots have been reported for microbial pigment and bioproducts fermentations when conventional electricity grid mixes are considered (Mussagy et al. 2020).

An exception was observed for the mineral resource scarcity category, in which dilute-acid hydrolysis emerged as the main contributor. This impact is primarily associated with sulfuric acid consumption, which affects the availability of critical minerals such as copper (Cu) and molybdenum (Mo). Additionally, bran production and cell activation steps contributed up to approximately 30% of the total impact in certain categories.

Among the evaluated scenarios, RBH exhibited the best overall environmental performance across all impact categories. Although RBH did not present the lowest impacts during hydrolysate preparation, it enabled the highest concentration of carotenoids per unit volume of growth medium. As a result, the environmental burdens associated with fermentation (typically the most impactful stage) and cell activation were substantially reduced when impacts were normalized per mf of carotenoids produced.

SBH and WBH showed intermediate environmental performance, with comparable results across most impact categories. However, SBH performed less favorably in the mineral resource scarcity category due to the higher contribution of acid hydrolysis, which requires larger volumes of concentrated hydrolysate. Moreover, soybean bran production itself is associated with high environmental impacts related to phosphorus scarcity, reflecting intensive fertilization practices commonly employed in soybean cultivation.

CBH showed the lowest environmental impacts during hydrolysate preparation, as smaller volumes of concentrated hydrolysate were required to reach standardized sugar concentrations. Nevertheless, except for mineral resource scarcity, CBH exhibited the worst overall environmental performance. This outcome is primarily explained by its lower carotenoid productivity, which resulted in higher relative impacts from fermentation and cell activation when expressed per mg of carotenoids produced.

From an economic perspective, although a full techno-economic assessment was beyond the scope of this study, the cradle-to-gate LCA results provide valuable indirect insights into process feasibility by identifying the main environmental and energy hotspots associated with carotenoid production. In particular, the superior performance of RBH highlights important operational advantages, including the elimination of hydrolysate detoxification, nutrient supplementation, and acid recovery steps, which significantly reduce reagent consumption, energy demand, process complexity, and waste generation. These simplifications are expected to contribute to lower operational costs and improved industrial feasibility. Therefore, beyond demonstrating environmental benefits, the LCA results also reinforce the potential of the proposed strategy as a more sustainable and economically attractive approach for carotenoid bioproduction from agro-industrial residues.

In summary, the use of RBH emerges as the most environmentally and operationally advantageous option for yeast-based carotenoid production within a biorefinery context. These findings underscore the importance of feedstock selection in enhancing both the environmental and economic sustainability of bioprocesses, reinforcing the role of agro-industrial residue valorization in the development of circular and sustainable biotechnological production systems.

## Conclusions

This study demonstrates, for the first time, the potential of soybean, wheat, rice, and corn bran hydrolysates as low-cost and renewable substrates for carotenoid production by *Rhodotorula mucilaginosa*, with particular emphasis on rice bran hydrolysate (RBH) and soybean bran hydrolysate (SBH). The results clearly highlight the strong influence of hydrolysate composition on microbial metabolism, underscoring the importance of optimizing bioprocess conditions to simultaneously promote yeast growth and biopigment biosynthesis. Among the evaluated substrates, RBH exhibited the most favorable environmental performance, while CBH showed the highest overall environmental impact, except for the mineral resource scarcity category, in which SBH presented the greatest impact. These findings reinforce the relevance of feedstock selection in improving the environmental sustainability of microbial bioprocesses.

The proposed approach prioritizes sustainability by valorizing renewable agro-industrial by-products and avoiding additional processing steps, such as hydrolysate detoxification, nutrient supplementation, and acid recovery. By simplifying the process configuration, this strategy minimizes reagent consumption, reduces processing time, and prevents the generation of undesirable waste streams. Overall, the insights gained in this study contribute to the development of environmentally and economically sustainable bioprocesses to produce high-value compounds from renewable resources. Moreover, they open new avenues for future research on microbial carotenoid production and support the advancement of circular bioeconomy-driven resource management strategies.

## Supplementary Information

Below is the link to the electronic supplementary material.

Supplementary Material 1

## Data Availability

All data and materials will be available upon request.
