# Supplementary material for: Sustainable carotenoid production using amylaceous agro-industrial byproducts: process efficiency and environmental assessment
Source: Bioresour Bioprocess. 2026 Jun 13;13(1):91. doi: 10.1186/s40643-026-01086-5 (PMC13264648; doi:10.1186/s40643-026-01086-5)
Supplement: Supplementary file 1 — Supplementary Material 1 [file 40643_2026_1086_MOESM1_ESM.docx]

**Sustainable carotenoid production using amylaceous agro-industrial byproducts: Process efficiency and environmental assessment**

Thércia Rocha Balbino^a^, Salvador Sánchez-Muñoz^a^, Stephanie Custódio Inácio^a^, Gabriele Campelo Almeida^a^, Ana Cláudia Dias^b^, Júlio César Santos^c^, Silvio Silvério da Silva*^a^ and Jorge Fernando Brandão Pereira*^d^

1. Laboratory of Bioprocesses and Sustainable Products. Department of Biotechnology, Engineering School of Lorena, University of São Paulo (EEL-USP), 12.602.810. Lorena, SP, Brazil
2. Centre for Environmental and Marine Studies (CESAM), Department of Environment and Planning, University of Aveiro, Campus Universitário de Santiago, 3810-193 Aveiro, Portugal
3. Laboratory of Biopolymers, Bioreactors, and Process Simulation. Department of Biotechnology, Engineering School of Lorena, University of São Paulo (EEL-USP), 12.602.810. Lorena, SP, Brazil
4. University of Coimbra, CERES, Department of Chemical Engineering, Faculty of Sciences and Technology 3030-790 Coimbra, Portugal

****Corresponding author address:***

*a. Laboratory of Bioprocesses and Sustainable Products. Department of Biotechnology, Engineering School of Lorena, University of São Paulo (EEL-USP). Estrada Municipal do Campinho, s/n - Pte. Nova, Lorena - SP, 12602-810, Brazil*

*e-mail:* [*silviosilverio@usp.br*](mailto:silviosilverio@usp.br)

*ORCID: 0000-0003-0669-2784*

*d. University of Coimbra, CERES, Department of Chemical Engineering, Faculty of Sciences and Technology 3030-790 Coimbra, Portugal*

*e-mail:* [*jfbpereira@eq.uc.pt*](mailto:jfbpereira@eq.uc.pt)

*ORCID: 0000-0001-5959-0015*

**Figure S1.** Variation in cellular biomass (represented as the natural logarithm of Optical Density measured at 600nm – ln OD_600nm_) during 72h cultivation of *Rhodotorula mucilaginosa* in standardized hydrolysates: corn bran hydrolysate (CBH), soybean bran hydrolysate (SBH), rice bran hydrolysate (RBH), wheat bran hydrolysate (WBH).

**Table S1.** Global inventory of data by functional unit (1mg of carotenoids).

|  | Unit | CBH | SBH | RBH | WBH |
| --- | --- | --- | --- | --- | --- |
| *Input* | | | | | |
| Dilute-acid Hydrolysis | | | | | |
| Bran | g | 2.640 | 7.870 | 4.208 | 3.288 |
| H_2_SO_4_ | mL | 0.325 | 0.969 | 0.518 | 0.405 |
| Water | mL | 15.606 | 46.521 | 24.877 | 19.437 |
| Electricity | Kwh | 0.001 | 0.002 | 0.001 | 0.001 |
| Filtration | | | | | |
| Electricity | Kwh | 0.002 | 0.005 | 0.003 | 0.002 |
| Preparation of standardized hydrolysates | | | | | |
| Water | mL | 81. 611 | 0.000 | 13.061 | 32.313 |
| Cell activation | | | | | |
| Glucose | g | 2.929 | 1.252 | 1.071 | 1.516 |
| Peptone | g | 0.488 | 0.209 | 0.178 | 0.253 |
| Yeast Extract | g | 0.293 | 0.125 | 0.107 | 0.152 |
| Malt Extract | g | 0.293 | 0.125 | 0.107 | 0.152 |
| Electricity | Kwh | 0.005 | 0.002 | 0.002 | 0.002 |
| Cell concentration (centrifugation) | | | | | |
| Electricity | Kwh | 0.000 | 0.000 | 0.000 | 0.000 |
| Peptone | g | 0.001 | 0.000 | 0.000 | 0.000 |
| Water | mL | 0.781 | 0.334 | 0.286 | 0.404 |
| pH adjustment of standardized hydrolysate | | | | | |
| NaOH | g | 0.166 | 0.495 | 0.265 | 0.207 |
| Fermentation | | | | | |
| Electricity | Kwh | 0.202 | 0.086 | 0.074 | 0.104 |
| *Output* |  |  |  |  |  |
| Filtration | | | | | |
| Hydrolysate losses | mL | 2.276 | 6.784 | 3.628 | 2.834 |
| Fermentation | | | | | |
| Carotenoids | mg | 1.000 | 1.000 | 1.000 | 1.000 |
